# ZSM‐5 Zeolite Synthesis from Coal Fly Ash Synthesised Silica: Sole Silica & Alumina Source

**DOI:** 10.1002/open.202400314

**Published:** 2025-01-28

**Authors:** Thapelo Manyepedza, Emmanuel V. Gaolefufa, Isaac N. Beas, Manoko Maubane‐Nkadimeng, Moses T. Kabomo

**Affiliations:** ^1^ Department of Chemistry University of Botswana Botswana Private bag UB Gaborone 00704 Botswana; ^2^ Department of Natural resources and Materials Botswana Institute for Technology Research and Innovation Maranyane House Plot 50654 Machel Drive Gaborone Botswana; ^3^ Department of Chemical and Materials Engineering University of South Africa (UNISA) P/Bag X6, Florida Johannesburg 1710 South Africa; ^4^ Molecular Sciences Institute School of Chemistry University of the Witwatersrand Johannesburg 2050 South Africa; ^5^ DSI-NRF Centre of Excellence in Strong Materials University of the Witwatersrand Johannesburg 2050 South Africa; ^6^ Microscopy and Microanalysis Unit University of The Witwatersrand Johannesburg 2050 South Africa

**Keywords:** ZSM-5 zeolite synthesis, Coal fly ash, High-purity silica, Environmental sustainability, Microporous materials

## Abstract

This study explores the synthesis of ZSM‐5 zeolite using high‐purity mesoporous silica exclusively derived from coal fly ash (CFA), eliminating the need for additional silica or alumina sources. Traditional ZSM‐5 synthesis relies on costly and environmentally harmful pure chemicals, whereas this approach utilizes CFA, an industrial byproduct, addressing both cost and sustainability concerns. The synthesized ZSM‐5 zeolite demonstrates exceptional purity, with a surface area of 455.24 m^2^/g, and exhibits unique structural properties, confirmed through XRD, SEM, TEM, FTIR, TGA, and BET analyses. This method highlights the potential of CFA‐derived silica as a sustainable feedstock for zeolite production, promoting both environmental sustainability and cost‐effective industrial applications in catalysis, adsorption, and separation processes.

## Graphical abstract

This study developed ZSM‐5 zeolite using silica sourced from coal fly ash, eliminating the reliance on expensive pure chemicals. The innovative method yields high purity, 455.24 m^2^/g surface area, and unique properties, verified via advanced analyses. It highlights coal fly ash's potential for sustainable zeolite production, advancing eco‐friendly industrial applications.

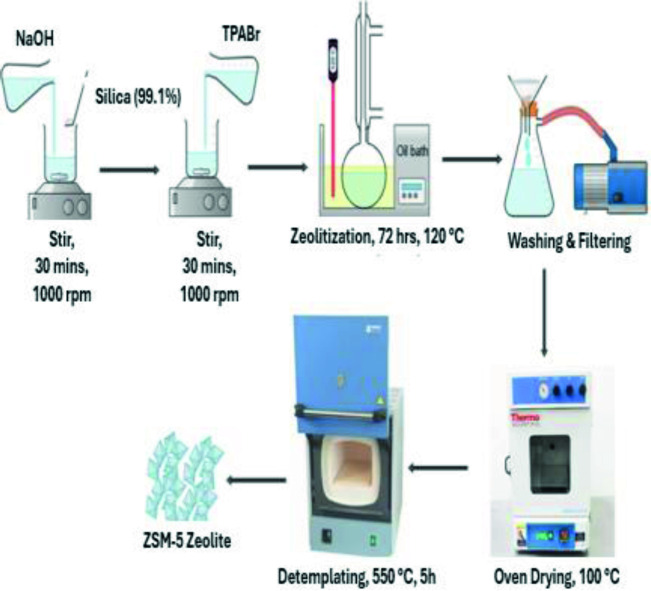

## Introduction

Zeolites, intricate aluminosilicate frameworks characterized by microporous structures of size (3–15 Å),[^1^, ^2^] possess distinctive properties pivotal for diverse industrial applications.^[3]^ These encompass Lewis acidity/basicity,[^4^, ^5^] expansive surface areas (200–900 m^2^ g^−1^),^[6]^ flexible porous architectures,[^4^, ^7^, ^8^] sieving capabilities,[^2^, ^9^, ^10^] and advantageous adsorption and ion‐exchange traits.^[6]^ Leveraging their unique structural topologies, zeolites find wide‐ranging utility across sectors including petroleum refining,[^11^, ^12^, ^13^, ^14^] wastewater treatment,[^15^, ^16^] gas purification,[^17^, ^18^] seawater desalination,^[19]^ soil enhancement,[^20^, ^21^] adsorbents, ion exchangers, and nanoparticle hosting.

Zeolite structures typically manifest as rigid, negatively charged, and microporous frameworks formed by TO_4_ tetrahedra sharing corners (T=Si, Al, P, or other tetrahedrally coordinated cations).[^22^, ^23^] These configurations facilitate the creation of channels and/or cavities with molecular dimensions, adept at accommodating inorganic or organic cations and water or solvent molecules, thereby neutralizing the framework's negative charge.[^24^, ^25^] While the exchangeability of inorganic cations is limited, organic cations can be eliminated via combustion, leaving protons embedded within the zeolite.[^6^, ^26^] Moreover, applying heat to expel water or solvent molecules helps preserve the framework structure. The enhanced pore accessibility of other molecules is achieved through the calcined state of zeolites, attained via heat treatment.^[27]^

ZSM‐5, a notable zeolite variant, has garnered significant attention owing to its unique channel topologies, notably the Mobil‐Five (MFI) type and high‐silica pentasil, boasting an opening capacity ranging from approximately 5.1–5.6 Å.^[6]^ With exceptional selectivity, hydrophobic characteristics, robust acid resistance, and thermal stability, ZSM‐5 proves highly adaptable for diverse applications in the realms of catalysis and adsorption.[^28^, ^29^, ^30^]

The synthesis of ZSM‐5 zeolites largely involves using several pure compounds as silicon (Si) and aluminium (Al) sources. The chemicals utilised in the synthesis process include tetraethyl orthosilicate (TEOS) (Si(OC_2_H_5_)_4_),[^31^, ^32^] fumed silica,^[33]^ Na_2_SiO_3_/Na_2_SiO_3_.xH_2_O,[^32^, ^34^, ^35^] Ludox‐AS‐40 colloidal sol (SiO_2_ 40 wt %),^[32]^ NaAlO_2_, Al(NO_3_)_3_.9H_2_O, and Al_2_(SO_4_)_3_.18H_2_O.[^6^, ^36^, ^37^] Each of these chemicals plays a role in the synthesis process.^[38]^

Direct ZSM‐5 zeolite synthesis using pure chemicals is costly and environmentally damaging, especially in large‐scale production.[^6^, ^39^] Interzeolite transformation offers a promising alternative for ZSM‐5 synthesis, where parent zeolites disintegrate or dissolve to release building units, rearranging to form new zeolites.[^40^, ^41^, ^42^] However, this process often requires harsh conditions, like high alkalinity/acidity, potentially decreasing product yield due to elevated silicon concentration in the liquid.[^42^, ^43^, ^44^] Utilizing cost‐effective raw materials for ZSM‐5 zeolite synthesis presents economic advantages over traditional methods reliant on pure chemicals, mitigating costs and environmental impact.[^39^, ^45^, ^46^, ^47^, ^48^]

According to numerous studies, the synthesis route typically necessitates an additional source of silica or alumina to achieve the production of high‐purity ZSM‐5 zeolite. Two studies conducted by Missengue[^46^, ^49^] synthesized ZSM‐5 zeolite using coal fly ash. While no new sources of alumina or silica were added in the other study, fumed silica^[50]^ was added as an additional source of silica in one trial. In Pertiwi's^[50]^ study, ZSM‐5 catalyst was synthesized from coal fly ash as a source of alumina and silica, supplemented with rice husk ash for additional silica, using seeded ZSM‐5. Krisnandi^[47]^ synthesized ZSM‐5 zeolite from coal fly ash, adjusting the Si/Al ratio with a solution of rice husk. Chareonpanich^[51]^ produced ZSM‐5 from coal fly ash and rice husk, initially obtaining only zeolite P due to low Si/Al ratios. Adjusting the Si/Al ratio with sodium silicate solution led to a 59 % weight yield of ZSM‐5. Feng and co.^[52]^ investigated direct synthesis of ZSM‐5 from coal fly ash as the sole source of silica and alumina. They found that an additional silica source, TEOS, was necessary for ZSM‐5 zeolite crystallization.

In Ndlovu's groundbreaking research,^[53]^ an innovative alkaline leaching method was employed to extract low purity untreated fly ash silica extract (coded UFSE) (27.28 % SiO_2_ and 0.21 % Al_2_O_3_, with 71.32 % Na attributed to NaOH added during alkaline fusion), from coal fly ash (CFA). The resulting solid residue underwent transformation into sodalite zeolite. Furthermore, the extracted silica underwent subsequent treatment with water, resulting in UFSE‐H_2_O (68.32 % SiO_2_ and 0.61 % Al_2_O_3_) with a notable reduction in Na content by 57.75 % to 30.13 %, enhancing its suitability for synthesizing high‐silica zeolites such as ZSM‐5 with a Si/Al ratio >10. This water‐washed/treated silica served as the exclusive source for synthesizing pure‐phase ZSM‐5, eliminating the requirement for additional aluminosilicate sources. This approach showcases the remarkable potential of CFA‐derived silica in zeolite production.

This study demonstrates a significant advancement by successfully synthesizing ZSM‐5 zeolite using high‐purity (99.1 % Si) mesoporous silica extracted directly from coal fly ash, diverging from conventional processes that typically require precursors with noticeable amounts of silica and alumina content. Remarkably, this process eliminates the need for additional treatments to adjust the Si/Al ratio typically required for zeolite synthesis, showcasing a more efficient and sustainable approach to producing ZSM‐5. Serving as the exclusive source of silica and alumina, this silica precursor enables the synthesis of a ZSM‐5 zeolite characterized by both exceptional purity, yield, and a significantly large surface area. By utilizing industrial waste materials in this innovative manner, the study not only contributes to the production of high‐quality ZSM‐5 zeolite but also promotes environmental sustainability. Due to its exceptional purity and surface properties, the resulting zeolite exhibits immense potential for diverse industrial applications, including catalysis, adsorption, and separation processes. As a result, this study represents a major advancement in the synthesis of zeolites and highlights the feasibility of using alternative resources to generate sustainable materials.

## Materials and Methods

The mesoporous silica utilized in this study was synthesized using the method outlined in our previous work.^[54]^ Chemicals employed in the study, including NaOH and tetrapropylammonium bromide (TPABr), were sourced from Sigma‐Aldrich and possessed a purity of 98 %. De‐ionized water served as the solvent for all experiments.

### Synthesis of ZSM‐5 Zeolite

A total of 12.04 g of high‐purity mesoporous silica (99.1 % SiO_2_, 0.1 % Al_2_O_3_) was mixed with 100 mL of distilled water containing 2.96 g NaOH, then stirred at 1000 rpm for 30 minutes. This solution was then gradually combined, in 5 mL increments, with a 100 mL tetrapropylammonium bromide (TPABr) solution under continuous stirring. The mixture was refluxed at 120 °C for 72 hours to form ZSM‐5 precipitate. The resulting solid was washed with distilled water to a neutral pH (7), then dried overnight at 100 °C. Finally, the dried ZSM‐5 was detemplated by heating at 550 °C for 10 hours.

### Characterization of ZSM‐5 Zeolite

ZSM‐5 zeolite characterization was performed using several analytical techniques. Powder X‐ray diffraction (XRD) measurements were conducted on a PANalytical XPert Pro X‐ray diffractometer with a CuKα X‐ray source (wavelength 1.5405 Å). Diffraction patterns were recorded over a 2θ range of 10° to 80° with the X‐ray detector operating at 40 kV and 40 mA. Prominent peaks were identified post‐scan and compared to patterns in the software library, selecting the one with the highest percentage match. Morphological analysis of the samples was carried out with a high‐resolution scanning electron microscope (Carl Zeiss Gemini SEM500). For high‐resolution transmission electron microscopy (HRTEM) imaging of ZSM‐5 zeolite, a Tecnai G2 30ST was used, operating at 200 kV. Functional groups were identified using a ThermoFisher Scientific Nicolet iS50 FTIR spectrometer over a spectral range of 400–4000 cm^−1^. Thermal stability was assessed with a Mettler Toledo TGA/DSC3+ thermogravimetric analyzer coupled to a Hiden Analytical Mass Spectrometer (HPR‐20 EGA) under argon flow (40 mL/min) from 25 °C to 900 °C to monitor volatile product evolution. Surface area and pore size distribution were measured with a Micromeritics 3Flex BET analyzer. Samples (40–60 mg) were degassed under vacuum (10^−5^ torr) at 100 °C for 1 hour, followed by overnight degassing at 250 °C before analysis.

## Results and Discussion

### Characterization of Silica from our Previous Study

**Figure 1:**
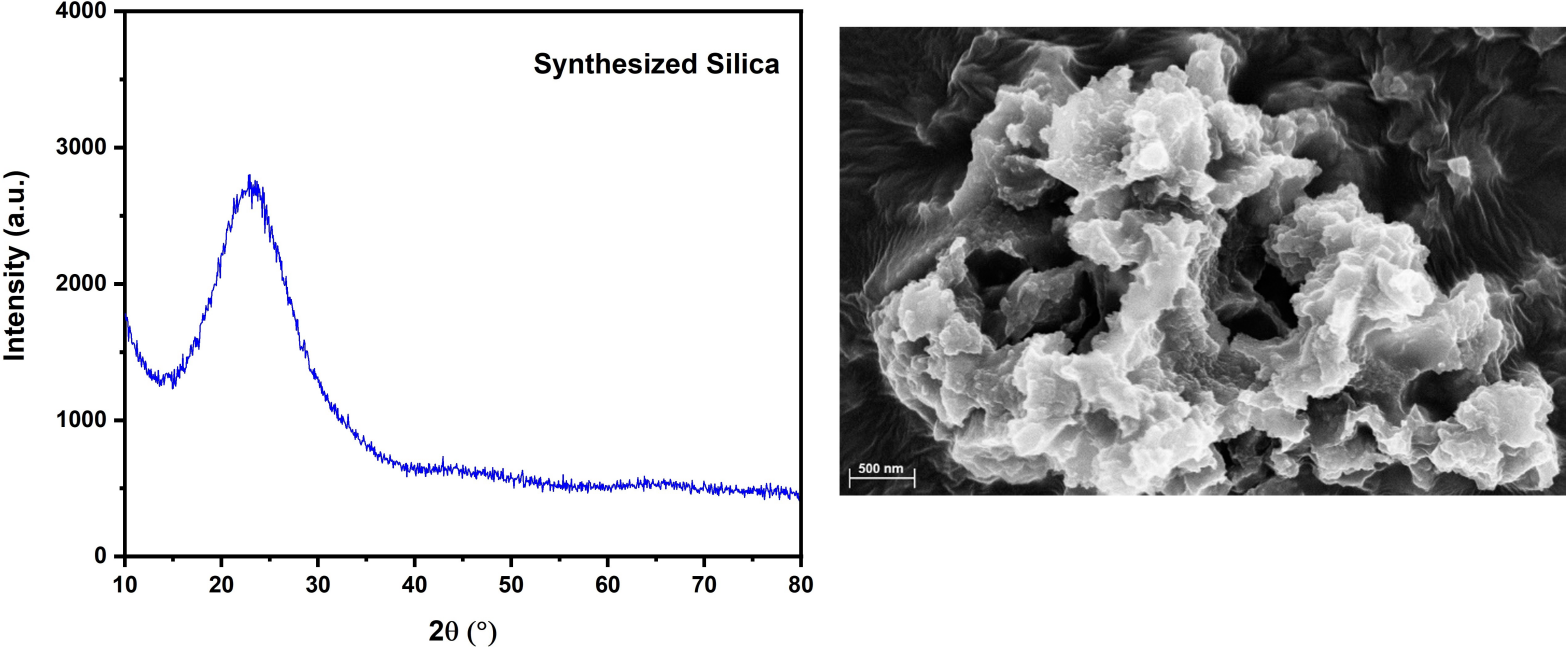
XRD diffractogram and SEM image of Silica synthesized from coal fly ash.^[54]^ “Copyright 2024, John Wiley & Sons. Distributed under terms and conditions of Creative Commons Attribution (CC BY) license (https://creativecommons.org/licenses/by/4.0/)”.

The X‐ray diffraction (XRD) spectrum and scanning electron microscope (SEM) image in Figure 1 demonstrate the amorphous nature and lack of regular structure in the silica produced from coal fly ash. The synthesized silica exhibits a high purity of 99.1 %, with minimal quantities of additional elements such as TiO_2_ (0.3 %), Fe_2_O_3_ (0.2 %), Al_2_O_3_ (0.1 %), Cl (0.1 %), and ZrO_2_ (0.1 %).^[54]^ This exceptional purity, combined with its non‐crystalline form, makes it an excellent precursor for the synthesis of high‐silica zeolites like ZSM‐5.^[55]^ The inclusion of a small amount of alumina (Al_2_O_3_) is particularly advantageous, as it provides a necessary source of aluminum for the structural framework of ZSM‐5 zeolite.[^49^, ^55^, ^56^] According to the Lowenstein rule, two aluminum atoms cannot be adjacent in the tetrahedral positions of the molecular sieve framework.^[57]^ Therefore, a higher Si/Al ratio or the use of very high‐purity silica in the crystallization synthesis system promotes the formation of the crystal nucleus of a ZSM‐5 molecular sieve. Overall, the structure and properties of this synthesized silica indicate significant potential for the efficient production of ZSM‐5 zeolite.

### Characterization of ZSM‐5

**Figure 2:**
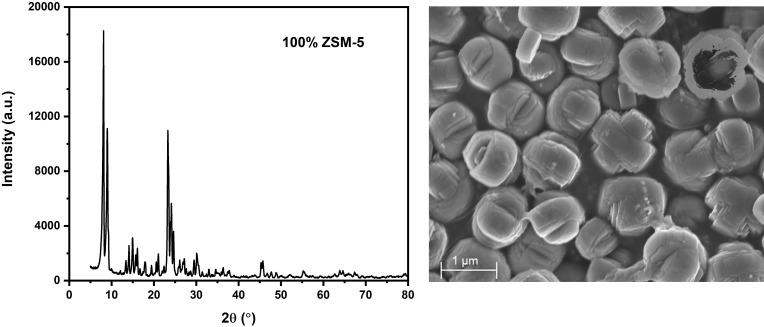
XRD diffraction and SEM pattern for the synthesised ZSM‐5 zeolite.

Figure 2 below displays the XRD spectrum of the synthesized ZSM‐5 zeolite, demonstrating the complete transformation of high‐purity silica from coal fly ash into ZSM‐5 zeolite. The broad peak between 20° and 30° observed in pure silica disappears, replaced by distinct crystalline peaks indicative of ZSM‐5 formation. The spectrum reveals a highly crystalline structure with no amorphous regions, characterized by prominent ZSM‐5 peaks at 2θ values of 8.086°, 8.923°, 14.046°, 14.882°, 15.928°, 23.246°, 24.083°, 25.965°, 27.010°, 29.415°, and 30.147°. These 2θ readings align closely with those reported in previous studies by Nguyen,^[57]^ Missengue,^[49]^ Zheng (2023),^[58]^ Kordatos,^[59]^ Sari^[60]^ and Krisnandi,^[47]^ confirming the successful synthesis and high crystallinity of the ZSM‐5 zeolite. The absence of any amorphous phase in the XRD spectrum underscores the effectiveness of the synthesis process in converting coal fly ash‐derived silica into a pure and well‐ordered ZSM‐5 zeolite structure.

The SEM image reveals well‐formed crystals, corroborating the high crystallinity of ZSM‐5 as indicated by the sharp XRD peaks. Figure 2 shows a distinct morphological structure, characterized by cross‐penetrative solid ovals with a plus‐like cross‐sectional view, which differs in texture from the Boron‐ZSM‐5 synthesized by Dai^[61]^ using a seed‐induction approach with chemical reagents. This comparison highlights the unique crystal morphology of the ZSM‐5 synthesized in this study, suggesting potential variations in synthesis conditions, such as the source of silica or the crystallization process, which can significantly influence the textural and structural properties of the resulting zeolite. The observed differences in crystal texture could be attributed to the specific synthesis parameters employed, which may affect nucleation and growth kinetics, leading to distinct crystal habits and degrees of crystallinity.

### TEM

**Figure 3:**
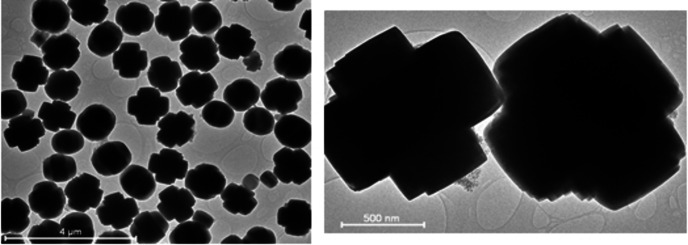
HRTEM images of the synthesised ZSM‐5 zeolite.

The Scanning Electron Microscopy (SEM) morphology depicted in Figure 2 is further validated by the Transmission Electron Microscopy (TEM) images presented in Figure 3. The TEM images provide a detailed view of the synthesized crystalline ZSM‐5, demonstrating its uniform structure. These images collectively confirm the consistency and homogeneity of the ZSM‐5 crystals, thereby reinforcing the observations made from the SEM analysis. The complementary use of both SEM and TEM techniques provides a comprehensive characterization of the ZSM‐5 morphology and crystallinity, ensuring the reliability of the synthesis process.

### FTIR

**Figure 4:**
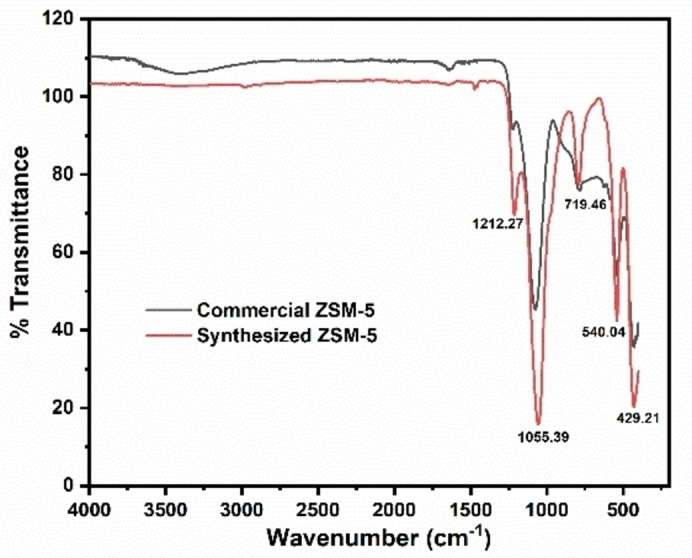
The FTIR of the synthesised ZSM‐5 zeolite mapped onto the commercial zeolite.

The unique vibrational modes of the Si−O−Si and Al−O−Si frameworks within the ZSM‐5 zeolite structure are represented by wavebands at 1212.27 cm^−1^, 1055.39 cm^−1^, 719.46 cm^−1^, 540.04 cm^−1^, and 429.21 cm^−1^ in Figure 4. The band at 1212.27 cm^−1^ is commonly linked to the asymmetric stretching vibrations of internal Si−O−Si bonds,[^59^, ^62^, ^63^] indicating robust and organized connections within the zeolite framework. The band at 1055.39 cm^−1^ is associated with asymmetric stretching vibrations of Si−O−Si, potentially influenced by Si−O−Al bonds, reflecting the overall network connectivity.[^64^, ^65^] The 719.46 cm^−1^ band corresponds to symmetric stretching vibrations of Si−O−Si bonds, providing insights into the regularity and homogeneity of the tetrahedral framework. The 540.04 cm^−1^ band is attributed to double‐ring vibrations or symmetric bending vibrations of Si−O−Si linkages, indicative of secondary building units such as double five‐membered rings (D5R) confirming the presence of the MFI (Mobil‐type Five) structure.[^58^, ^59^, ^62^, ^65^, ^66^] Lastly, the 429.21 cm^−1^ band is associated with bending vibrations of Si−O−Si bonds, indicating the flexibility and stability of the framework at lower frequencies.^[58]^

These combined vibrational bands help identify the structural characteristics and verify the integrity of the ZSM‐5 zeolite framework, ensuring that the synthesis has successfully produced the intended crystalline structure. This is also demonstrated by the perfect match between the synthesized ZSM‐5 and the commercial ZSM‐5 peaks, with the notable exception of the broad ‐OH band at 3250–3750 cm^−1^, corresponding to the hydroxyl group's stretching vibration.^[67]^ This difference is attributed to the presence of Si−OH and Al−OH groups on the ZSM‐5 surface, as well as ‐OH groups from adsorbed water. The variation arises from the detemplating process in the synthesized ZSM‐5, which removes most of the hydroxyl groups associated with water.

### TGA

**Figure 5:**
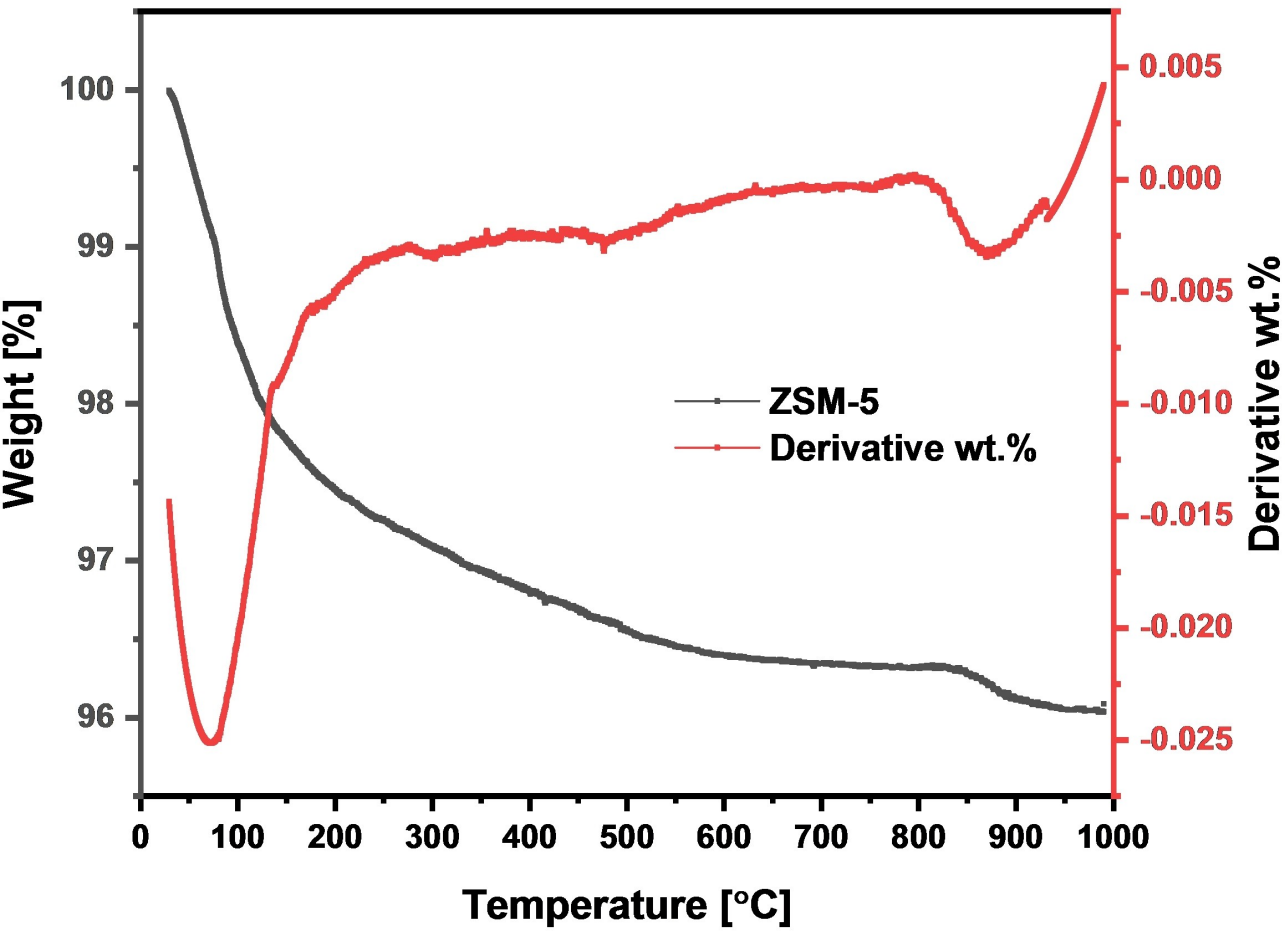
TGA analysis of the synthesised ZSM‐5 zeolite.

The thermogravimetric analysis (TGA) plot in Figure 5 of TPABr‐detemplated ZSM‐5 zeolite, synthesized using silica derived exclusively from coal fly ash as the source of both silica and alumina, shows significant mass losses at three specific temperature ranges.

Previous reports have also confirmed that aluminosilicates typically exhibit three distinct weight loss regions during thermal treatment.[^68^, ^69^] The initial mass reduction of approximately 1 % occurs within the 50–100 °C range, likely due to the evaporation of adsorbed water (physisorbed or interlayer adsorbed water molecules).[^68^, ^70^] A further mass loss of 2.62 % is observed between 100–600 °C, attributed to the decomposition and removal of organic template residues.[^58^, ^71^] Beyond this, the plot exhibits a consistent plateau at 600 °C, indicating the thermal stability of the synthesised ZSM‐5 zeolite framework and condensation and dehydration of structural hydroxyl groups.[^72^, ^73^] A minor mass loss of around 0.05 % between 850–1000 °C suggests the removal of any residual impurities or further stabilization of the zeolite structure. These observations confirm the effective detemplation process and demonstrate the synthesized ZSM‐5 zeolite's capacity to maintain structural integrity at high temperatures.[^57^, ^74^]

### BET

**Figure 6:**
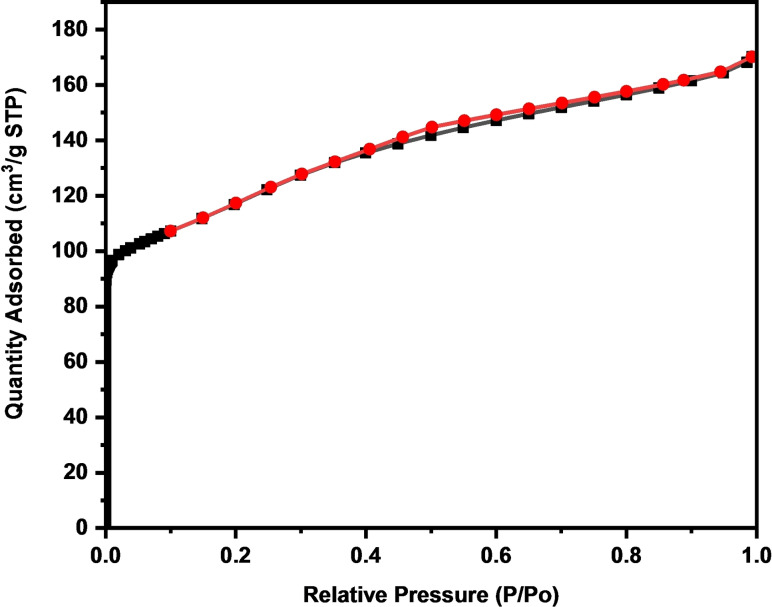
N_2_ adsorption‐desorption isotherms of the synthesised ZSM‐5 zeolite.

Nitrogen adsorption/desorption isotherms were measured using a surface area analyzer based on the BET principle to confirm the porous nature of the synthesized silica. The adsorption‐desorption isotherm shown in Figure 6 for the synthesized ZSM‐5 zeolite predominantly exhibits characteristics of a Type I isotherm, which is typically observed in microporous materials with small external surfaces.[^75^, ^76^, ^77^] Examples of such materials include certain activated carbons, molecular sieve zeolites, and specific porous oxides.^[78]^ However, the presence of an H4 hysteresis loop at higher relative pressures (P/P0 values ranging from 0.5–0.7) indicates a deviation from the classic Type I isotherm. This hysteresis loop is a distinctive feature of Type IV isotherms, which are associated with materials possessing both microporosity and mesoporosity.^[79]^ Type IV isotherms are characterized by the sequential adsorption of monolayers and multilayers, followed by capillary condensation that leads to the complete filling of the pores.[^78^, ^80^, ^81^]

Thus, the observed isotherm suggests that the synthesized ZSM‐5 zeolite exhibits a combination of micro and mesoporous structures. This dual porosity significantly enhances the material's potential applications. The micropores contribute a high surface area and adsorption capacity, while the mesopores improve diffusion properties.

Previous studies have explored various methods for synthesizing ZSM‐5 zeolite from coal fly ash (CFA). Missengue^[49]^ used oxalic acid‐treated CFA with alumina content ranging from 1.9 % to 29.3 %, achieving surface areas of 353–459 m^2^/g. The variation in surface areas was attributed to the presence of unreacted fly ash. Additionally, the high amounts of alumina, as high as 29 %, could have led to the formation of other crystalline types of zeolites,^[82]^ potentially reducing the purity of the ZSM‐5. In another study, Missengue^[46]^ synthesized highly microporous ZSM‐5 from CFA with added fumed silica, obtaining a surface area of 328 m^2^/g. Pertiwi^[50]^ synthesized ZSM‐5 from CFA without templates, using commercial ZSM‐5 as a seed and rice husk ash as an additional silica source, resulting in a surface area of 114.87 m^2^/g.

In contrast, our study utilized high‐purity silica (99.1 % Si and 0.1 % Al) derived from CFA as the sole source of both silica and alumina for synthesizing ZSM‐5. This approach allows for precise control over aluminum distribution within the zeolite framework, minimizing impurities and enhancing the crystalline structure.[^83^, ^84^] The controlled introduction of aluminum stabilizes the zeolite structure thermodynamically and optimizes crystallization kinetics,[^82^, ^85^] leading to uniform crystals as evidenced by SEM (Figure 2) and TEM (Figure 3) images. Our method resulted in 100 % crystalline ZSM‐5 with a notable surface area of 455.24 m^2^/g and a pore size of 3.97 nm (*see comparison with other studies in* Table 1), as supported by the BET isotherm plot in Figure 4, which shows both micropores and mesopores.[^78^, ^79^] The high‐purity silica used in our study contributed to the formation of a more uniform and well‐defined zeolite structure, enhancing the packing density of the framework and increasing the concentration of accessible active sites.[^86^, ^87^, ^88^] This approach is groundbreaking as no other study, to our knowledge, has utilized pure silica from CFA in this manner, offering a significant improvement over previous methods in terms of crystalline quality and surface area.

**Table 1:** Comparison of ZSM‐5 zeolites synthesised from other studies.

	Material Synthesis			Material Characterization		
Source	Material	Amount of Alumina in Starting Material (%)	Additional Silica or Alumina Source	Surface Area (m^2^/g)	Pore Size (nm)	References
High Purity Silica**	ZSM‐5	0.1	N/A	455.24	3.97	This work
CFA	ZSM‐5	1.9–29	N/A	353–459	N/A	[49]
CFA	ZSM‐5/Sodalite	N/A	ZSM‐5 seed	114.87	9.41	[50]
CFA	ZSM‐5	24.6	Fumed Silica	328	N/A	[46]
CFA	ZSM‐5	N/A	Rice Husk	43.76	26.26	[47]
CFA	ZSM‐5	10.5	Tetraethyl Orthosilicate	358.5	N/A	[52]
CFA	ZSM‐5	32.55	Rice Husk	321.19	3.84	[83]

**High purity silica from reference.^[54]^

This study demonstrates the feasibility of achieving a large surface area and excellent purity in ZSM‐5 zeolite using silica derived exclusively from coal fly ash as the primary source of silica and alumina. The synthesized ZSM‐5 exhibited significant surface area and purity, both crucial for its potential industrial applications. The synthesized ZSM‐5 possesses a substantial surface area, confirmed by BET surface area measurements, and features adjustable pore sizes, enhancing its suitability for a wide range of industrial applications. BET isotherm analysis reveals the presence of both micropores and mesopores, which broaden its potential applications in catalysis and adsorption. Micropores contribute to increased surface area and adsorption capacity, while mesopores facilitate efficient mass transfer and diffusion within the material. ZSM‐5 synthesized from coal fly ash‐derived silica presents properties that position it as a highly promising material for applications requiring high surface area, purity, and controlled pore structure. Potential industrial uses include harnessing its adsorption and catalytic capabilities in catalytic converters, gas separation processes, and environmental remediation technologies.

## Conclusions

This study demonstrates a novel and sustainable approach to synthesizing high‐purity ZSM‐5 zeolite using mesoporous silica exclusively derived from coal fly ash (CFA), eliminating the need for additional silica or alumina sources. The synthesized ZSM‐5 exhibits remarkable structural integrity, high surface area (455.24 m^2^/g), and purity, validated through various characterization techniques. This method addresses the environmental and economic drawbacks associated with traditional synthesis using pure chemicals and leverages industrial waste, promoting environmental sustainability. The resulting ZSM‐5 zeolite holds significant potential for diverse industrial applications, including catalysis, adsorption, and separation processes, marking a significant advancement in zeolite synthesis technology.

1

## Conflict of Interests

The authors declare no conflict of interest.

## Data Availability

Data is available upon reasonable request.

## References

[1] E. M. Gallego , C. Paris , M. R. Díaz-Rey , M. E. Martínez-Armero , J. Martínez-Triguero , C. Martínez , M. Moliner , A. Corma , Chem. Sci. 2017, 8, 8138–8149. 29568462 10.1039/c7sc02858j PMC5855293

[2] J. Wen , H. Dong , G. Zeng , J. Clean Prod. 2018, 197, 1435–1446.

[3] L. B. McCusker , Ch. Baerlocher , E. Jahn , M. Bülow , Zeolites 1991, 11, 308–313.

[4] N. Kosinov , C. Liu , E. J. M. Hensen , E. A. Pidko , Chem. Mater. 2018, 30, 3177–3198. 29861546 10.1021/acs.chemmater.8b01311 PMC5973782

[5] A. Rodríguez-Fernández , J. R. Di Iorio , C. Paris , M. Boronat , A. Corma , Y. Román-Leshkov , M. Moliner , Chem. Sci. 2020, 11, 10225–10235. 34094288 10.1039/d0sc03809a PMC8162407

[6] D. Nguyen , V. Dinh , H. Q. Nguyen , N. T. Hung , J. Chem. Technol. Biotechnol. 2023, 98, 1339–1355.

[7] V. Verdoliva , M. Saviano , S. De Luca , Catalysts 2019, 9, 248.

[8] I. Khan , X. Chu , I. Khan , H. Liu , W. Li , L. Bai , L. Jing , Mater. Res. Bull. 2020, 130, 110926.

[9] H. Yang , Y. Qiao , Z. Chang , H. Deng , X. Zhu , R. Zhu , Z. Xiong , P. He , H. Zhou , Adv. Mater. 2021, 33, DOI: 10.1002/adma.202102415. 34338385

[10] R. Xiong , J. Chen , L. Zhang , P. Li , X. Yan , Y. Song , W. Luo , T. Tang , G. Sang , M. Hirscher , Microporous Mesoporous Mater. 2021, 313, 110820.

[11] P. Lu , J. Sun , D. Shen , R. Yang , C. Xing , C. Lu , N. Tsubaki , S. Shan , Appl. Energy 2018, 209, 1–7.

[12] P. Zhang , G. Yang , L. Tan , P. Ai , R. Yang , N. Tsubaki , Catal. Today 2018, 303, 77–85.

[13] S. Watcharasing, C. Wattanakit, S. Salakhum, A. Prasertsab, P. Kiattikomol, in *Day 3 Thu, March 24*, *2022*, OTC, **2022**. Virtual and Kuala Lumpur.

[14] L. Hong , J. Zang , B. Li , G. Liu , Y. Wang , L. Wu , Inorganics (Basel) 2023, 11, 214.

[15] E. C. Umejuru , T. Mashifana , V. Kandjou , M. Amani-Beni , H. Sadeghifar , M. Fayazi , H. Karimi-Maleh , N. T. Sithole , Environ. Res. 2023, 231, 116073. 37164282 10.1016/j.envres.2023.116073

[16] M. V. Obuzdina , E. A. Rush , RUDN J. Ecol. Life Safety 2022, 30, 240–249.

[17] K. Qi , L. Gao , X. Li , F. He , Catalysts 2023, 13, 855.

[18] S. Abbasi , M. R. Khosravi-Nikou , A. Shariati , Chem. Eng. Process 2023, 184, 109294.

[19] K. Meng , X. Li , Y. Niu , C. Zhang , X. Yu , J. Rong , H. Hou , H. Chen , Phys. Chem. Chem. Phys. 2023, 25, 16908–16920. 37325848 10.1039/d3cp00787a

[20] J. Szerement , K. Jurek , J. Mokrzycki , R. Jarosz , P. Oleszczuk , M. Mierzwa-Hersztek , Soil Tillage Res. 2023, 230, 105701.

[21] V. GirijaVeni , K. S. Reddy , K. L. Sharma , K. S. Shankar , J. Rohit , in Soil Science: Fundamentals to Recent Advances, Springer Singapore, Singapore 2021, pp. 449–467.

[22] A. Ghorbanpour , A. Gumidyala , L. C. Grabow , S. P. Crossley , J. D. Rimer , ACS Nano 2015, 9, 4006–4016. 25824422 10.1021/acsnano.5b01308

[23] A. Khaleque , M. M. Alam , M. Hoque , S. Mondal , J. Bin Haider , B. Xu , M. A. H. Johir , A. K. Karmakar , J. L. Zhou , M. B. Ahmed , M. A. Moni , Environ. Adv. 2020, 2, 100019.

[24] N. Widiastuti , H. Wu , H. M. Ang , D. Zhang , Desalination 2011, 277, 15–23.

[25] A. Farkaš , M. Rožić , Ž. Barbarić-Mikočević , J. Hazard Mater. 2005, 117, 25–33. 15621350 10.1016/j.jhazmat.2004.05.035

[26] Q. Zhang , S. Gao , J. Yu , Chem. Rev. 2023, 123, 6039–6106. 36049046 10.1021/acs.chemrev.2c00315

[27] J. H. Park , K. S. Sin , S. Chang , S. H. Park , S. J. Cho , Catal. Today 2023, 411–412, 113866.

[28] B. Bensafi , N. Chouat , F. Djafri , Coord. Chem. Rev. 2023, 496, 215397.

[29] Zh. B. Budaev , A. A. Stepanov , L. L. Korobitsyna , A. V. Vosmerikov , Rev. Adv. Chem. 2023, 13, 53–59.

[30] D. Olson , J. Catal. 1980, 61, 390–396.

[31] G. T. M. Kadja , N. J. Azhari , R. R. Mukti , M. Khalil , ACS Omega 2021, 6, 925–933. 33458544 10.1021/acsomega.0c05070 PMC7808162

[32] Y.-J. Wang , J.-P. Cao , X.-Y. Ren , X.-B. Feng , X.-Y. Zhao , Y. Huang , X.-Y. Wei , Fuel 2020, 268, 117286.

[33] W. Luo , X. Yang , Z. Wang , W. Huang , J. Chen , W. Jiang , L. Wang , X. Cheng , Y. Deng , D. Zhao , Microporous Mesoporous Mater. 2017, 243, 112–118.

[34] R. Li , S. Chong , N. Altaf , Y. Gao , B. Louis , Q. Wang , Front. Chem. 2019, 7, DOI: 10.3389/fchem.2019.00505. PMC6647869 31380349

[35] Z. Chen , Z. Li , Y. Zhang , D. Chevella , G. Li , Y. Chen , X. Guo , J. Liu , J. Yu , Chem. Eng. J. 2020, 388, 124322.

[36] S. Anas Boussaa , D. Nibou , K. Benfadel , L. Talbi , A. Boukezzata , Y. Ouadah , D. Allam , Int. J. Comput. Experimen. Sci. Eng. 2023, 9, 156–160.

[37] O. Chen , S. C. Liu , P. Q. Zhang , S. Q. Zheng , Kemija u Industriji 2021, 70, 121–127.

[38] M. Wu , W. Jiang , J. Jiang , Y. Zou , P. Zhang , P. Mao , Y. Xu , Y. Shi , Bull. Mater. Sci. 2020, 43, 289.

[39] D. Azizi , F. Ibsaine , J. Dionne , L. C. Pasquier , L. Coudert , J. F. Blais , Microporous Mesoporous Mater. 2021, 318, 111029.

[40] L. Xu , Y. Yuan , Q. Han , L. Dong , L. Chen , X. Zhang , L. Xu , Catal. Sci. Technol. 2020, 10, 7904–7913.

[41] Y. Sun , C. Yang , Z. Wen , Z. Zhang , P. Wei , X. Wang , Q. Li , Sustain. Energy Fuels 2024, 8, 641–648.

[42] D. Shi , G. Fu , A. Omran , K.-G. Haw , L. Zhu , R. Ding , Q. Lang , S. Wang , Q. Fang , S. Qiu , X. Yang , V. Valtchev , Microporous Mesoporous Mater. 2023, 358, 112332.

[43] X. Li , S. Han , J. Xu , N. Jiang , Microporous Mesoporous Mater. 2023, 350, 112441.

[44] M. B. dos Santos , K. C. Vianna , H. O. Pastore , H. M. C. Andrade , A. J. S. Mascarenhas , Microporous Mesoporous Mater. 2020, 306, 110413.

[45] I. M. S. Anekwe , E. E. S. Lora , K. A. Subramanian , A. Kozlov , S. Zhang , B. Oboirien , Y. M. Isa , Energy Convers. Manage. X 2024, 22, 100529.

[46] R. N. M. Missengue , P. Losch , G. Sedres , N. M. Musyoka , O. O. Fatoba , B. Louis , P. Pale , L. F. Petrik , C. R. Chim. 2016, 20, 78–86.

[47] Y. K. Krisnandi , F. M. Yanti , S. D. S. Murti , IOP Conf. Ser. Mater. Sci. Eng. 2017, 188, 012031.

[48] S. Vichaphund , D. Aht-Ong , V. Sricharoenchaikul , D. Atong , Environ. Technol. 2014, 35, 2254–2261. 25145178 10.1080/09593330.2014.900118

[49] R. Missengue , P. Losch , N. Musyoka , B. Louis , P. Pale , L. Petrik , Catalysts 2018, 8, 124.

[50] A. Pertiwi, E. F. Destian, N. Valentino, S. I. Heryanti, G. N. Sevie, F. M. Yanti, A. Arfiana, A. Sholihah, H. Saputra, A. B. M. I. Syihab, Y. Yudatomo, S. D. S. Murti, **2023**, 2902, p. 030004.

[51] M. Chareonpanich , T. Namto , P. Kongkachuichay , J. Limtrakul , Fuel Process. Technol. 2004, 85, 1623–1634.

[52] R. Feng , K. Chen , X. Yan , X. Hu , Y. Zhang , J. Wu , Catalysts 2019, 9, 788.

[53] N. Z. N. Ndlovu , A. E. Ameh , L. F. Petrik , T. V. Ojumu , Mater. Today Commun. 2023, 34, 105436.

[54] T. Manyepedza, E. Gaolefufa, I. N. Beas, M. T. Kabomo, B. Modukanele, *ChemistrySelect* **2024**, *9*, e202305065.

[55] W. Qin , Y. Zhou , J. D. Rimer , React. Chem. Eng. 2019, 4, 1957–1968.

[56] Y. Ghrib , N. Frini-Srasra , E. Srasra , Surf. Eng. Appl. Electrochem. 2017, 53, 64–70.

[57] D. Nguyen , V. Dinh , H. Q. Nguyen , N. T. Hung , J. Chem. Technol. Biotechnol. 2023, 98, 1339–1355.

[58] Y. Zheng , J. Zhou , Z. Ma , X. Weng , L. Cheng , G. Tang , Materials (Basel) 2023, 16, 4338. 37374521 10.3390/ma16124338 PMC10305030

[59] K. Kordatos , S. Gavela , A. Ntziouni , K. N. Pistiolas , A. Kyritsi , V. Kasselouri-Rigopoulou , Microporous Mesoporous Mater. 2008, 115, 189–196.

[60] Z. G. L. V. Sari , H. Younesi , H. Kazemian , Appl. Nanosci. 2015, 5, 737–745.

[61] C. Dai , J. Li , A. Zhang , C. Nie , C. Song , X. Guo , RSC Adv. 2017, 7, 37915–37922.

[62] C. Zhang , S. Li , S. Bao , Waste Biomass Valorization 2019, 10, 2825–2835.

[63] R. M. Mohamed , H. M. Aly , M. F. El-Shahat , I. A. Ibrahim , Microporous Mesoporous Mater. 2005, 79, 7–12.

[64] B. Karakaya Yalçin , B. İpek , Turk. J. Chem. 2020, 44, 841–858. 33488198 10.3906/kim-2001-42 PMC7671202

[65] I. Ali , A. Hassan , S. Shabaan , K. El-Nasser , Arabian J. Chem. 2017, 10, S2106–S2114.

[66] Y.-P. Guo , H.-J. Wang , Y.-J. Guo , L.-H. Guo , L.-F. Chu , C.-X. Guo , Chem. Eng. J. 2011, 166, 391–400.

[67] R. Sabarish , G. Unnikrishnan , J. Porous Mater. 2020, 27, 691–700.

[68] O. B. Ayodele , Sci. Rep. 2017, 7, 10008. 28855545 10.1038/s41598-017-09706-z PMC5577191

[69] S. Jiang , H. Zhang , Y. Yan , X. Zhang , RSC Adv. 2015, 5, 41269–41277.

[70] S. Hassanpour , M. Taghizadeh , F. Yaripour , Ind. Eng. Chem. Res. 2010, 49, 4063–4069.

[71] M. S. M. Kamil , K. K. Cheralathan , J. Porous Mater. 2020, 27, 587–601.

[72] O. B. Ayodele , H. F. Abbas , W. M. A. W. Daud , Ind. Eng. Chem. Res. 2014, 53, 650–657.

[73] J. Cao , R. Hou , F. Wang , M. Xing , Y. Han , Y. Guo , K. Hao , W. Zheng , L. Zhang , Z. Tao , X. Wen , H. Xiang , Y. Yang , Y. Li , Catal. Lett. 2024, DOI: 10.1007/s10562-024-04704-z.

[74] N. Precisvalle , M. Mancinelli , M. Ardit , G. Beltrami , L. Gigli , A. Aloise , E. Catizzone , M. Migliori , G. Giordano , V. Guidi , A. Martucci , Crystals (Basel) 2023, 13, 979.

[75] C. Zhao , X. Hu , C. Liu , D. Chen , J. Yun , X. Jiang , N. Wei , M. Li , Z. Chen , J. Environ. Chem. Eng. 2022, 10, 106868.

[76] R. Z. Kuvatova , O. S. Travkina , B. I. Kutepov , Catal. Ind. 2021, 13, 99–104.

[77] O. Chen , S. C. Liu , P. Q. Zhang , S. Q. Zheng , Kemija u Industriji 2021, 70, 121–127.

[78] M. Thommes , K. Kaneko , A. V. Neimark , J. P. Olivier , F. Rodriguez-Reinoso , J. Rouquerol , K. S. W. Sing , Pure Appl. Chem. 2015, 87, 1051–1069.

[79] K. Y. Nandiwale , A. M. Pande , V. V. Bokade , RSC Adv. 2015, 5, 79224–79231.

[80] F. Mikšík , T. Miyazaki , K. Thu , Energies (Basel) 2020, 13, 4247.

[81] C. Buttersack , Phys. Chem. Chem. Phys. 2019, 21, 5614–5626. 30788465 10.1039/c8cp07751g

[82] S. Hong , A. J. Mallette , J. J. Neeway , R. K. Motkuri , J. D. Rimer , G. Mpourmpakis , Dalton Trans. 2023, 52, 1301–1315. 36625388 10.1039/d2dt02764j

[83] Z. Sun , Q. Shu , Q. Zhang , S. Li , G. Zhu , C. Wang , J. Zhang , H. Li , Z. Huang , Separations 2024, 11, 39.

[84] A. W. Budiman , K. D. Nugrahaningtyas , S. D. P. W. Purnama , A. K. Nabila , P. I. Gerard , D. W. T. Wulansari , A. I. Sabiilagusti , R. I. Arvianto , J. Phys. Conf. Ser. 2022, 2190, 012003.

[85] S. Inagaki , N. Yamada , M. Nishii , Y. Nishi , Y. Kubota , Microporous Mesoporous Mater. 2020, 302, 110223.

[86] E. Pérez-Botella , M. Palomino , G. B. Báfero , H. O. Pastore , S. Valencia , F. Rey , J. CO2 Util. 2023, 72, 102490.

[87] T. G. Sours , A. R. Kulkarni , J. Phys. Chem. C 2023, 127, 1455–1463. 10.1021/acs.jpcc.2c08429 PMC9885523 36733763

[88] D. S. Wragg , R. E. Morris , A. W. Burton , Chem. Mater. 2008, 20, 1561–1570.

